# The Role of Inflammation in Age-Related Sarcopenia

**DOI:** 10.3389/fphys.2017.01045

**Published:** 2017-12-12

**Authors:** Sebastiaan Dalle, Lenka Rossmeislova, Katrien Koppo

**Affiliations:** ^1^Exercise Physiology Research Group, Department of Kinesiology, KU Leuven, Leuven, Belgium; ^2^Department for the Study of Obesity and Diabetes, Third Faculty of Medicine, Charles University, Prague, Czechia

**Keywords:** muscle wasting, muscle protein metabolism, NSAID, sarcopenia, protein supplementation, resistance training, inflammation

## Abstract

Many physiological changes occur with aging. These changes often, directly or indirectly, result in a deterioration of the quality of life and even in a shortening of life expectancy. Besides increased levels of reactive oxygen species, DNA damage and cell apoptosis, another important factor affecting the aging process involves a systemic chronic low-grade inflammation. This condition has already been shown to be interrelated with several (sub)clinical conditions, such as insulin resistance, atherosclerosis and Alzheimer's disease. Recent evidence, however, shows that chronic low-grade inflammation also contributes to the loss of muscle mass, strength and functionality, referred to as sarcopenia, as it affects both muscle protein breakdown and synthesis through several signaling pathways. Classic interventions to counteract age-related muscle wasting mainly focus on resistance training and/or protein supplementation to overcome the anabolic inflexibility from which elderly suffer. Although the elderly benefit from these classic interventions, the therapeutic potential of anti-inflammatory strategies is of great interest, as these might add up to/support the anabolic effect of resistance exercise and/or protein supplementation. In this review, the molecular interaction between inflammation, anabolic sensitivity and muscle protein metabolism in sarcopenic elderly will be addressed.

## Introduction

Aging is generally associated with numerous changes that may, directly or indirectly, affect health and/or life span. One of the major problems in the aging population is a progressive loss in skeletal muscle mass, muscle strength, and/or functionality, described as age-related sarcopenia. The elderly suffering from this multifactorial pathological condition are at risk of adverse outcomes such as physical disability, injuries, frailty, social exclusion, hospitalization and eventually an increased mortality (Cruz-Jentoft et al., 2010; Visser and Schaap, 2011). Several strategies to attenuate the loss of muscle mass and other muscle impairments that comes with aging have been developed (Sakuma and Yamaguchi, 2012; Denison et al., 2015). However, none of these have been proven successful to fully reverse the muscle wasting condition. Given the high prevalence of sarcopenia in the aging population and the associated high health care costs, it is of importance to reveal and elucidate the working mechanisms which underlie muscle protein metabolism in the elderly, in order to optimize the classic interventions and/or to develop new ones.

Muscle protein metabolism is carefully regulated by counterbalanced fluctuations in muscle protein breakdown (MPB) and muscle protein synthesis (MPS) (Churchward-Venne et al., 2014). In elderly, the balance between MPB and MPS seems to be disturbed, which progressively increases the loss of skeletal muscle mass (Churchward-Venne et al., 2014). Many underlying factors such as hormonal changes, decreased activity, diminished nutrient intake, and neuronal changes were reported in the literature (Dirks et al., 2006; Budui et al., 2015), but lately, the role of inflammation on the regulation of muscle protein metabolism has gained more and more interest among gerontologists. Generally, aging is associated with a chronic state of slightly increased plasma levels of pro-inflammatory mediators, such as tumor necrosis factor α (TNFα), interleukin 6 (IL-6) and C-reactive protein (CRP) (for an overview see Table 1). This state is often referred to as a low-grade inflammation (LGI) and is, at least partly, the manifestation of increased numbers of cells leaving the cell cycle and entering the state of cellular senescence. Indeed, senescent cells acquire a Senescence-Associated Secretory Phenotype, which induces the production of pro-inflammatory cytokines (TNFα, IL-6 and an overactivation of NF-κB) (Tchkonia et al., 2013). Moreover, there is a growing interest in the association between the telomere/telomerase system and LGI, as cellular senescence can be triggered by critically short telomeres, representing irreparable DNA damage (Kordinas et al., 2016). Also, there are indications that LGI can directly cause telomere/telomerase dysfunction, enforcing the vicious LGI circle and stimulating an accelerated aging phenotype (Jurk et al., 2014).

**Table 1:** Studies reporting plasma/serum levels of pro-inflammatory markers with aging.

**Marker**	**Study**	**Main finding**
TNFα	Bruunsgaard et al., 2000	↑ plasma [TNFα] in elderly (81 years) vs. young adults (19–31 years)
	Bruunsgaard et al., 1999	↑ plasma [TNFα] in elderly (>80 years) (2.5 pg/ml) vs. young adults (18–30 years) (1.4 pg/ml)
	Paolisso et al., 1998	Significant correlation between age and TNFα (*r* = 0.64)
	Paik et al., 2013	↑ serum [TNFα] in elderly ♀ (60–79 years) (>9 pg/ml) vs. younger ♀ (30–59 years) (±7 pg/ml)
IL-6	Wei et al., 1992	↑ plasma [IL-6] in elderly (55–75 years) ♂ vs. younger (26–54 years) ♂ and significant correlation between age and IL-6 (*r* = 0.28)
	Hager et al., 1994	Significant correlation between age and IL-6 (*r* = 0.47)
	McKane et al., 1994	Significant correlation between age and IL-6 in ♀ (*r* = 0.48)
	Checouich et al., 1995	Significant correlation between age and IL-6 in ♀ (*r* = 0.75)
	Cohen et al., 1997	↑ mean log(IL-6) and median IL-6 levels in elderly >90 years vs. elderly 80–89 years vs. elderly 70–79 years
	Harris et al., 1999	↑ log(IL6) values in elderly >80 years (0.96 ± 0.65 pg/ml) vs. 71–72 years (0.73 ± 0.66 pg/ml)
	Ferrucci et al., 2005	↑ plasma [IL-6] in elderly >75 years (>1.4 pg/ml) vs. young adults (20–39 years) (0.6 pg/ml)
hs-CRP	Ferrucci et al., 2005	↑ plasma [CRP] in elderly >75 years (>2.6 mg/l) vs. young adults (20–39 years) (1.0 mg/l)
	Puzianowska-Kuznicka et al., 2016	↑ plasma [CRP] in elderly >80 years (>2.4 mg/l) vs. elderly 65–69 years (2.2 mg/l)
	Paik et al., 2013	↑ serum [CRP] in elderly ♀ (>50 years) (>1.1 mg/l) vs. younger ♀ (30–59 years) (±0.95 g/l)

*TNFα, tumor necrosis factor α; IL-6, interleukin 6; hs-CRP, high-sensitive C-reactive protein*.

Although it has been suggested that inflammatory mediators affect muscle protein metabolism (Frasca and Blomberg, 2016), it is not fully understood to what extent and through which signaling pathways they induce muscle wasting. Population-based data suggest that circulating concentrations of IL-6 and TNFα are significantly elevated in sarcopenic elderly (Bian et al., 2017) and it was reported that higher IL-6 and CRP levels increase the risk of muscle strength loss (Schaap et al., 2006). In a 10-year longitudinal study in community-dwelling elderly, plasma concentrations of TNFα, IL-6, and IL-1 were shown to be strong predictors of morbidity and mortality in older subjects (Baylis et al., 2013). Furthermore, systemic inflammation was also reported as one of the primary mediators of skeletal muscle wasting in diseases such AIDS, COPD, chronic heart failure and cancer (Sakuma et al., 2015) and it was shown to accelerate aging in general (Jurk et al., 2014). Without pronouncing on causality, these findings suggest that there is a link between inflammatory mediators and muscle mass and function.

This review is particularly concerned with the recent progress that has been made in understanding the role of inflammation in age-related sarcopenia and in muscle protein metabolism in general. In the first sections, pathways of both MPB and MPS will be described and related to inflammation. The following sections will give an overview of the classic approaches (i.e., aerobic/resistance exercise, protein supplementation) that attempt to attenuate age-related sarcopenia. Finally, interventions that target the administration of compounds with anti-inflammatory properties (vitamin D, poly-unsaturated fatty acids, non-steroidal anti-inflammatory drugs) will be discussed as an alternative approach.

## Muscle protein breakdown and its regulation by inflammation

As mentioned above, elevated levels of pro-inflammatory plasma cytokines and acute-phase proteins are often observed in sarcopenic elderly. Despite strong correlations between inflammatory markers and the risk for functional decline and mortality in elderly, conclusive evidence on a causative link between these markers and age-related sarcopenia is hard to establish (Reuben et al., 2003; Schaap et al., 2006). Recent evidence, however, suggests that chronic LGI is, at least partially, involved in the onset and/or progression of age-related muscle wasting (Beyer et al., 2012; Argilés et al., 2015). Higher levels of inflammatory markers were reported to negatively affect skeletal muscle metabolism through direct catabolic or indirect mechanisms, such as decreases in growth hormones (Sakuma et al., 2015). Though, the challenge remains to further uncover the specific molecular mechanisms through which inflammation interacts with muscle protein metabolism.

Acute inflammation models are widely applied to study the molecular mechanisms which link inflammation and muscle protein metabolism. In rodents, the effect of lipopolysaccharide injection has been shown to induce muscle catabolism through inflammatory signaling (Schakman et al., 2012). Accordingly, blunting an acute infection-induced inflammation improved muscle performance in elderly (Beyer et al., 2011). However, it should be noted that acute high-grade inflammation has a limited translational potential compared to the LGI present in the aged population.

In general, proteolysis is regulated through four main pathways (Combaret et al., 2009; Fanzani et al., 2012; Bowen et al., 2015). The contribution of each pathway is context- (e.g., stress vs. basal) or tissue- (e.g., brain vs. muscle) dependent (de Oliviera Nunes Teixeira et al., 2012). Their mechanisms are also inter-dependent and might co-regulate proteolysis on a different hierarchical level (Dirks et al., 2006). In the following section, the four main proteolytic pathways (ubiquitin proteasome, calpains, macro autophagy, apoptosis) are briefly discussed, as well as their interaction(s) with inflammatory signaling.

### ATP-dependent ubiquitin-proteasome pathway

The ATP-dependent ubiquitin-proteasome pathway is responsible for degrading the majority of proteins (80–90%) (Lilienbaum, 2013). The proteolytic core of the 26S complex, a 20S proteasome, degrades ubiquitin-conjugated proteins after binding with a regulatory 19S particle. This conjugation to ubiquitin marks abnormal (misfolded, denaturated) or short-lived proteins for rapid hydrolysis (Rock et al., 1994). Ubiquitination of specific substrates is closely regulated by ubiquitin protein (E3) ligases, such as MuRF1 and MAFbx, specifically in muscle cells (Bodine and Baehr, 2014). Evidence indicates that the expression of both E3 ligases is upregulated in several models of muscle atrophy, such as immobilization, denervation, hind limb unloading, dexamethasone treatment, and IL-1-induced cachexia (Bodine and Baehr, 2014). The role of the ubiquitin-proteasome pathway in sarcopenia, however, is less clear (Bowen et al., 2015; Fan et al., 2016; Wilson et al., 2017). Some studies reported elevated levels of MAFbx and MuRF1 in aged rat muscle (Clavel et al., 2006; Altun et al., 2010), while others observed decreased levels of atrogenes with aging (Erik et al., 2017). Likewise, in human studies, some authors reported no differences in skeletal muscle protease activity between different age groups (Bossola et al., 2008), while others noticed higher mRNA levels of proteins encoding protease components, ubiquitin-like proteins and proteins related to ubiquitin function in elderly compared to younger adults (Welle et al., 2003). Furthermore, elevated levels of the ubiquitin protein in old human muscles mainly occurred in fast-twitch muscles, such as *extensor digitorum longus*, while no differences were detected in the slow-twitch *soleus* muscle (Cai et al., 2004). Dissimilarities between studies might therefore be attributed to the studied muscle type, as well as the specific study population, as it was suggested that age-related muscle wasting is mechanistically different from muscle wasting-related confounders such as disuse, bedrest or disease (Erik et al., 2017). A recent review suggested that elevated levels of pro-inflammatory mediators (due to LGI), such as TNFα and IL-6, might upregulate this proteolytic pathway through activation of FOXO3a, which regulates the ubiquitin-proteasome system (Xia et al., 2017). It remains to be elucidated, however, whether low levels of these pro-inflammatory mediators are (on the long-term) sufficient to induce this proteolytic pathway. To conclude, the ubiquitin-proteasome pathway is likely to be involved in the regulation of muscle wasting with aging, partially due to its upregulation with inactivity. On the other hand, findings on the association between LGI and the ubiquitin-proteasome pathway are scarce and need to be further elucidated.

### Calpains

Calpains are a family of cysteine proteases with a wide range of cellular calcium-regulated functions. They are responsible for the proteolysis of several substrates, including cytoskeletal and membrane proteins, enzymes and transcription factors (Dargelos et al., 2008). Furthermore, calpains are also involved in apoptosis through two major pathways, triggered by cell death signals (TNF) or endoplasmatic reticulum (ER) stress, respectively (Dargelos et al., 2008). Reactive oxygen species (ROS) accumulation with advanced age is able to upregulate calpain activity directly (Nakashima et al., 2004) or indirectly by inducing Ca^2+^ release from the ER or oxidation of MAPK-dependent pathways (Kefaloyianni et al., 2006). Although the evidence is scarce, the contribution of calpains to age-related muscle wasting is not excluded. Higher calpain availability and activity, and lower availability of its endogenous inhibitor (calpastatin), were observed in muscles of old (23 months) compared to young rats (3 months) and treatment of dystrophic muscle with calpain inhibitors attenuated muscle degeneration (Dargelos et al., 2007). It was reported that intracellular [Ca^2+^] were elevated in aged rodent muscle cells, potentially contributing to an increased calpain activation with aging (Andersson et al., 2011). To our knowledge, there are no data which compare calpain acitivity/availability in old vs. young human muscles. Calpains can also promote inflammation through several mechanisms leading to NF-κB (pro-inflammatory transcription factor) activation and the production of pro-inflammatory cytokines (Ji et al., 2016). However, it is unclear whether calpains are involved in muscle proteolysis through regulation of inflammation in aged muscles. Therefore, more studies are required to further elaborate the mechanistic association between calpains and age-related muscle wasting in humans.

### (Macro)autophagy pathway

The (macro)autophagy pathway is as a housekeeping mechanism responsible for the clearance of dysfunctional organelles and damaged macromolecules (e.g., protein aggregates). The autophagosome, a double-membrane vesicle, encloses macromolecules or organelles for delivery to the lysosome, in which hydrolases stand in for the degradation into recycled “building blocks,” e.g., amino acids. The lysosomal protease cathepsin L was shown to be involved in muscle proteolysis upon acute infection (Deval et al., 2001). Both *in vitro* and *in vivo* studies reported that elevated levels of the pro-inflammatory cytokine IL-6 is a first mechanism which links inflammation to cathepsin-induced muscle atrophy (Ebisui et al., 1995; Tsujinaka et al., 1995). Whether cathepsin L plays a crucial role in age-related muscle wasting is less straightforward, as higher levels of cathepsin L were observed in the *soleus* muscle of old (30 months) vs. young (3 months) rats (Pattison et al., 2003), while no different expression patterns were found in the *gastrocnemius* muscle of old (30 months) and young (3 months) rats (O'Connell et al., 2007).

Autophagic activity in the fast-twitch *plantaris* muscle decreases with aging, resulting in insufficient clearance and accumulation of intracellular waste products, such as lipofuscin, protein aggregates and damaged mitochondria (Wohlgemuth et al., 2010). In general, autophagy is accepted to be a promising target to counteract sarcopenia (Fan et al., 2016). Until now, physical exercise and caloric restriction (CR) were proven successful in preventing/attenuating the age-related decrease in autophagic activity. White et al. (2016) reported that long-term voluntary resistance wheel exercise in elderly mice increased basal autophagy (increased LC3III/I ratio, marker of autophagy) compared to sedentary age-matched controls, while MuRF1 and MAFbx remained unaffected. Another study found that a mild CR (8%) positively affected muscle composition, oxidative stress, cell death and autophagy in old rats, suggesting that mild CR might be an applicable intervention to combat age-related sarcopenia, given a sufficiently qualitative energy intake (Wohlgemuth et al., 2010). In human studies, markers of autophagy were downregulated in Duchenne muscular dystrophy patients when compared to healthy controls, suggesting a decreased autophagic activity in these patients (Palma et al., 2012). Although evidence is scarce, Jiao and Demontis (2017) suggested that comparable mechanisms may apply to the development of age-related muscle wasting in elderly.

Autophagy is also involved in the regulation of the production of inflammatory mediators upon acute inflammatory stimuli (e.g., LPS) (Cadwell, 2016). However, to our knowledge, a crosstalk between autophagy and LGI was not yet established in the literature. It is conceivable that inflammation contributes to a dysregulation of the autophagic/mitophagic activity by stimulation of oxidative stress (Li et al., 1999; Suematsu et al., 2003). Nevertheless, it remains to be clarified whether LGI is a sufficiently strong signal for inducing oxidative stress in muscle cells. Furthermore, upregulation of the classic NF-κB signaling is capable of inhibiting autophagy by suppression of autophagy-related genes (atg5 and beclin 1), but this mechanism was not confirmed yet in old skeletal muscle cells.

To summarize, skeletal muscle autophagy is downregulated with advanced aged and indirectly contributes to muscle wasting due to insufficient clearance of intracellular waste products or damaged organelles, such as mitochondria. An accumulation of damaged mitochondria and cellular waste generally induces oxidative stress, which enforces a pro-inflammatory environment in favor of catabolic processes.

### Apoptosis

Apoptosis is another main player contributing to the onset and progression of sarcopenia. Apoptosis, the process of programmed cell death, generally occurs during development and aging as a homeostatic mechanism to maintain cell populations in tissues (Elmore, 2007). Dirks and Leeuwenburgh (2002) reported higher levels of apoptosis (+50%) (measured by nucleosome fragmentation) in 24 months old rats compared to 6 months young rats. The same research group also looked at the mechanisms through which age-related apoptosis affected muscle wasting in skeletal muscles of old rats (Marzetti et al., 2008). They concluded that muscle weight declined progressively with advancing age, concomitant with increased apoptotic DNA fragmentation. A higher susceptibility of aged type II muscle fibers to TNFα-stimulated apoptotic signaling partially explains the greater loss of fast twitch muscle fibers with aging (Phillips and Leeuwenburgh, 2005).

In skeletal muscle, two main routes are described to induce apoptosis, i.e., an internal and an external pathway. In the (mitochondrial-dependent) internal pathway, non-receptor-mediated stimuli in the cytosol, such as elevated ROS and Ca^2+^ levels, can directly act upon mitochondrial homeostasis, increasing their permeability (Nitahara et al., 1998). Consequently, pro-apoptotic proteins are released from the intermembrane space into the cytosol. In the external pathway, ligands (such as TNFα) bind to their respective receptor and initiate the recruitment of adaptor proteins, eventually resulting in a death-induced caspase-cascade. As TNFα protein levels are generally elevated in both circulation and skeletal muscle in elderly (Greiwe et al., 2001), this pathway might also play a role in age-related muscle wasting. On the other hand, no apoptosis of muscle cells was observed in inflammatory myopathies, which questions the role of this pathway in age-related LGI (Migheli et al., 1997). Furthermore, it is not fully clear which apoptotic pathway is the main player in age-related muscle wasting. Future research should further reveal the contribution of each apoptotic pathway in order to develop targeted anti-apoptotic strategies. Similar to autophagy, exercise and mild CR were found to be effective in attenuating apoptosis with aging (Dirks and Leeuwenburgh, 2004; Phillips and Leeuwenburgh, 2005; Wohlgemuth et al., 2010). Despite the extensively studied association between age-related muscle wasting and apoptosis in rodents, evidence emerging from human samples are currently lacking to our knowledge. Therefore, human data studying the role of apoptosis in sarcopenia are required in order to see whether the mechanisms/observations are comparable to rodent models. As concluded by Marzetti and Bernabei (2012), targeting apoptosis might be an effective intervention to counteract age-related muscle wasting.

To conclude, several pathways are involved in the regulation of MPB in sarcopenia. However, it should be noted that possible other pathways might be active and remain to be confirmed in sarcopenic human muscles. These pathways contribute to muscle wasting to a certain extent, which depends on the unique context of each muscle fiber, i.e., fiber type, (im)mobility, oxidative damage, etc. Until now, no studies focused on the relative contribution of each proteolytic pathway. Therefore, future research should elucidate the contribution of each proteolytic pathway, taking into account the specific research sample/population/condition, in order to allow the development of effective and specific strategies, which target one or more proteolytic mechanisms.

## Muscle protein synthesis and its regulation by inflammation

Besides its putative role in MPB, inflammation negatively affects MPS. Already in 1984, Klasing and Austic (1984) established that high-grade inflammatory challenges, such as *E. coli* injection, decreased the *in vivo* MPS in broilers. Also, a more prolonged exposure to a high-grade inflammatory milieu, e.g., sepsis, reduced MPS, through decreased activation of the mTORC1 signaling pathway, and hence muscle mass, as reviewed by Frost and Lang (2011). The mechanistic link between low-grade inflammation and the downregulation of aged-related MPS, however, is less understood.

Muscle protein metabolism is the result of a balance between an increased post-prandial whole body protein synthesis and a decreased post-absorptive protein synthesis (Dardevet et al., 2012). This regulation is mainly orchestrated by the mTORC1 signaling pathway that integrates environmental stimuli to control cellular growth (Deldicque et al., 2005). In the muscle, many mTORC1 stimuli are described, including amino acids (leucine in particular), insulin, hormones released during mechanical stimuli as well as contractions *per se*, while energy deprivation and stress/hypoxia inhibit mTORC1 activity. Therefore, combined resistance exercise and protein supplementation has been proven effective in stimulating muscle growth in adults (Churchward-venne et al., 2012). Under basal conditions, MPS does not appear to be compromised in elderly, but its response to physiologic stimuli, such as amino acids, exercise or insulin (rather as a permissive mediator) is blunted (Dardevet et al., 2012; Drummond et al., 2012; Haran et al., 2012). This phenomenon is called “anabolic resistance” and implicates the need for higher intakes of amino acids and/or higher loads of resistance exercise, which is difficult to meet in a frail population that generally suffers from a loss of appetite (Rennie, 2009; Wysokinski et al., 2015).

### Protein intake as anabolic signal

#### Animal studies

Inflammation is an important underlying factor that contributes to the insensitivity to anabolic signals. The research group of Dardevet confirmed the interference of inflammatory background (NF-κB, TNFα, IL-6 etc.) with anabolic signaling in several animal studies. They observed that rats which developed LGI at 25 months were unresponsive, in their MPS, to food intake, while in rats without LGI MPS was significantly increased (Balage et al., 2010). Another study revealed that rats, whose LGI was attenuated by ibuprofen (NSAID) treatment, exhibited a restored protein anabolism at post-prandial state (Rieu et al., 2009). Similarly, the supplementation of an antioxidant mixture during 7 week, which reduced LGI, was effective in improving the anabolic response to leucine in skeletal muscles of aged rats (Marzani et al., 2008). In contrast, Mayot et al. (2008) observed no differences in MPS between aged-matched 24 months old LGI and no-LGI rats. However, the authors did not specifically mention when MPS was measured. Previous studies showed differences in post-prandial MPS with similar post-absorbative MPS between LGI and no-LGI aged rats (Rieu et al., 2009; Balage et al., 2010).

#### Human studies

To our knowledge, only two human studies looked at the association between LGI and meal-induced stimulation of MPS. A study executed by the research group of Dardevet found that elderly with LGI (assessed by increased CRP levels) had no different post-absorptive or post-prandial MPS, compared to elderly without LGI (Buffière et al., 2015). The authors suggested that other pro-inflammatory cytokines (TNFα, IL-1/6) might be more suitable to establish the association between LGI and anabolic resistance. Furthermore, the post-protein bolus time interval during which MPS is measured was 5 h in this study, while other studies recommend a shorter period of 1.5–2 h (Bohé et al., 2001; Norton et al., 2009; Atherton et al., 2010; Wilson et al., 2011). Similarly, it was found that ibuprofen (1,800 mg.d^−1^) administration during 1 week in elderly with LGI did not affect MPS in response to a whey protein bolus when compared to placebo (Dideriksen et al., 2016). However, this might be due to the lack of efficacy of a short NSAID administration period of 1 week, demonstrated by the lack of decrease in CRP levels in this group. Furthermore, it is possible that the anabolic effect of anti-inflammatory strategies (e.g., NSAIDs) might partially occur via a decreased MPB rather than an increased MPS. Future human trials are warranted in order to reproduce the findings of earlier animal studies in which the association between LGI and anabolic resistance was established (Marzani et al., 2008; Rieu et al., 2009; Balage et al., 2010). The studies should take into account the exposure time to LGI, the treatment duration, applied LGI biomarker(s), sufficiently large sample sizes, and the post-prandial time interval to assess MPS.

### Exercise as anabolic signal

Besides the effect of inflammation on the anabolic response to food/protein intake, its effect on the anabolic response to exercise is broadly studied. These findings might also contribute to our understanding of why some elderly gain less muscle mass with relatively identical exercise stimuli compared to younger adults. Interesting to note is that NSAID administration in younger adults vs. elderly results into different adaptations to exercise training. In younger adults, studies which looked at the acute effects of NSAID intake on the muscle response to a single bout of resistance exercise observed a decrease in MPS compared to placebo (Trappe et al., 2002) and an attenuated satellite cell proliferation up to 8 days after resistance exercise (Mikkelsen et al., 2009) or endurance exercise (Mackey et al., 2007). Also, when long-term (8 week) resistance training was combined with a daily high-dose (1,200 mg.d^−1^) of ibuprofen intake, muscle strength and muscle hypertrophic adaptations to resistance training were impaired compared to a control condition (Lilja et al., 2017). In contrast, a moderate dose of ibuprofen (400 mg.d^−1^) was not sufficient to negatively affect muscle strength and hypertrophy following 6 week resistance training (Krentz et al., 2008). Similar to the results observed in younger adults, a study performed in young rats reported that ibuprofen administration (~20 mg.kg^−1^.d^−1^) during chronic overload by synergist ablation for 14 days inhibited muscle hypertrophy with ~50% (Soltow et al., 2006). The authors, however, were not sure whether the inhibition of hypertrophy by the NSAID was caused by its interference with the regeneration process (decreased satellite cell activity), an attenuation of MPS, or both. A downregulated MPS response to resistance exercise with NSAID intake could be ascribed to a blunted prostaglandin F_2α_ (PGF_2α_) increase, which normally stimulates skeletal MPS (Rodemann and Goldberg, 1982).

Contrary to the younger adults, long-term resistance training combined with NSAID intake, unexpectedly induced additional gains in muscle mass and/or muscle strength in elderly (Trappe et al., 2011) and osteoarthritis patients (Petersen et al., 2011). Trappe et al. (2011) speculated that COX inhibition, an important mechanism of action of NSAIDs, might have a relatively stronger inhibitory effect on MPB compared to MPS in elderly. Besides blunting PGF_2α_, it was also shown that COX inhibition might suppress protein degradation by reducing intramuscular production of prostaglandin E_2_ (PGE_2_), eventually resulting in a higher net protein balance (Rodemann and Goldberg, 1982). It cannot be excluded that the effect of NSAID administration on training adaptations is partly related to its pain-relieving feature, as it was reported that osteoarthritis patients were able to produce higher maximal strengths after 12 week of ibuprofen (1,200 mg.d^−1^) administration (Petersen et al., 2011).

In general, it is important to interpret findings concerning the role of inflammation in muscle protein metabolism upon exercise with caution, taking into account the study population (adults vs. elderly with LGI/patient populations), type of exercise/muscle adaptation (endurance vs. resistance exercise vs. muscle damage following severe resistance exercise), and the time span of evaluation of muscle effects (immediately following exercise vs. long-term effects). Furthermore, studies should focus on mechanisms through which MPS is affected by LGI. Currently, many studies do not or only superficially report findings concerning the COX or prostaglandin pathways.

## Satellite cells and their regulation by inflammation

Satellite cells are adults muscle stem cells which play an important role in muscle growth and repair (Yin et al., 2013). Under basal conditions, satellite cells remain sublaminal in a quiescent state. Upon muscle damage, satellite cells exit their quiescent state, start to proliferate through the sarcolemma and fuse with existing muscle fibers. These processes are accompanied by specific expression patterns of myogenic regulator factor (MRF) genes and protein levels. Adult quiescent satellite cells express Pax7, while Myf5 and/or MyoD expression is rapidly upregulated following satellite cell activation, both regulated by Pax 7 (Cornelison and Wold, 1997; Rudnicki et al., 2008).

The lower regenerative potential of aged muscles can be explained by a deterioration in satellite cell differentiation and a reduced Pax7 pool of myogenic stem cells (Collins et al., 2007; Bernet et al., 2014). Furthermore, recent evidence suggests that many aged satellite cells switch from the quiescent state to an irreversible senescence state, and fail to activate and expand upon injury (Sousa-Victor et al., 2014). The reduced satellite cell function with aging might be due to altered systemic factors which affect satellite cell activity and differentiation (Conboy et al., 2005), such as altered Notch signaling in muscles, altered circulating levels of protein growth differentiation factor 11, reduced levels of IGF-1, increased inflammation and pro-inflammatory cytokines (Harridge, 2003; Degens, 2010; Jang et al., 2011; Sinha et al., 2014). It was shown that TNFα, in particular, reduces the expression of MyoD and myogenin in myoblasts and destabilizes MyoD in regenerating mice muscles (Szalay et al., 1997; Langen et al., 2004). Therefore, it can be hypothesized that some effects of LGI on muscle protein metabolism are mediated through changes in satellite cell function, as TNFα levels affect MRF expression.

## Classic approaches targeting age-related sarcopenia

Many studies currently focus on developing strategies to combat age-related sarcopenia. In this regard, life style interventions are of great interest due to their relatively straightforward applicability. These interventions can be subdivided in two main categories, i.e., exercise and nutrition. Both aerobic and resistance exercise, as well as the supplementation of amino acids/proteins, vitamin D (vit D) and polyunsaturated fatty acids (PUFAs), have ergogenic implications on the regulation of muscle mass in elderly. In addition, aerobic exercise, and the supplementation of vit D or PUFAs showed interesting interactions with the modulation of inflammation, and might therefore decrease LGI and subsequently reduce muscle wasting in elderly.

### Aerobic exercise

Aerobic exercise has been suggested as a meaningful strategy to combat age-related sarcopenia. Besides its effects on cardiovascular fitness and endurance, aerobic exercise is a potent inducer of muscle size and strength (Konopka and Harber, 2014). Recent evidence indicates that endurance exercise-induced increases in muscle size and strength are due to an increased MPS, similarly in elderly and young adults (Harber et al., 2012; Ozaki et al., 2013; Konopka and Harber, 2014). Some studies concluded that gains in muscle growth, induced by aerobic exercise, are comparable to the hypertrophic response observed following resistance training, and that endurance exercise can be considered an effective countermeasure for muscle loss with advancing age (Konopka and Harber, 2014).

The mechanisms, which underlie these positive effects on the muscle hypertrophic response, are diverse. Firstly, an upregulation of the transcription of genes involved in mitochondrial biogenesis, such as CAMK, AMPK, and PGC1α (Iolascon et al., 2014), increases the muscle mitochondrial content and function. This eventually results in an attenuated production of mtROS and thus oxidative stress, regarded as a potential mediator in skeletal muscle loss with aging (Jackson, 2016). Secondly, aerobic exercise training also has an impact on MPB, as basal FOXO3a (upstream of MAFbx and MuRF1) and myostatin (inhibitor of MPS upstream of Akt-mTORC1) mRNA levels were reduced in elderly following a 12 week training program, with concomitant muscle hypertrophy (Konopka et al., 2010). Another indirect mechanism involves the partial alleviation of age-related insulin resistance, which contributes to the age-related anabolic resistance to protein supplementation (Dickinson et al., 2014; Konopka and Harber, 2014). Finally, aerobic exercise also exhibits anti-inflammatory capacities (Montero-Fernandez and Serra-Rexach, 2013). Transient elevations of IL-6, released in response to acute exercise, might stimulate in the long-term the expression of anti-inflammatory mediators (such as IL-1 receptor antagonist and IL-10) and downregulate the expression of pro-inflammatory mediators (such as TNFα and IL-1β) (Pedersen et al., 2007). Together, these findings indicate that long-term aerobic training is effective to overcome anabolic resistance and decrease MPB, exhibiting a protective effect on muscle wasting.

### Resistance exercise

Despite these promising findings, resistance exercise is generally accepted as the most effective approach to induce muscle hypertrophy in both young and old adults, as the relative gain in muscle size in response to resistance training was found to be similar in adult (<65 years) and young individuals (Narici et al., 2004; Slivka et al., 2008; Raue et al., 2009).

The hypertrophic response following resistance training is mediated through several mechanisms. The mechanical stimuli *per se* and growth factors released in response to mechanical stimuli are both able to independently activate the mTORC1 pathway. The activation of mTORC1 by growth factors (insulin-like growth factor, mechano growth factor) occurs via the “classic” PI3K/Akt signaling (Bodine et al., 2001). Mechanical stimuli have a more acute effect, which is, contrarily, not always dependent on PI3K/Akt signaling (Hornberger et al., 2004). It was suggested that mechanical signals can activate the mTORC1 pathway after they are converted in biological responses. The proposed mediators of these stimuli are phosphatidic acid, which is highly produced in response to stretching and activates p70S6K, and integrins, which connect the extracellular matrix to the muscle cell membrane (Zanchi and Lancha, 2008).

Despite positive findings in adults (<65 years), Peterson et al. (2011) reported less pronounced gains in lean body mass in response to resistance exercise in elderly. This indicates that the myocellular response to resistance training is blunted in advanced age (Narici et al., 2004; Slivka et al., 2008; Raue et al., 2009). The precise mechanism(s), underlying the development of this anabolic insensitivity in elderly, are not fully understood yet. It is likely, however, that the limited activation of the mTORC1 axis following an acute bout of exercise in elderly is an important contributor to this observation (Kumar et al., 2009; Fry et al., 2011). Accordingly, one study found an impaired downregulation of REDD1 (potent inhibitor of mTORC1) mRNA in elderly following resistance exercise (Greig et al., 2011). Nevertheless, it is conceivable that also inflammation is somehow involved in the blunted adaptation to resistance exercise, since the effect of resistance training on gains in muscle mass and muscle strength was reinforced when combined with NSAID administration in elderly (Trappe et al., 2011).

### Protein supplementation

Among the nutritional interventions, supplementation with proteins/amino acids (leucine in particular) is widely applied to treat muscle loss in elderly. Proteins stimulate MPS, and are therefore generally expected to attenuate muscle wasting. The effects of protein supplementation on MPS are mediated through several mechanisms. Firstly, protein synthesis is induced by cell swelling *per se* (Lang et al., 1993). This swelling can be obtained through an increased cellular osmolarity, due to Na^+^-dependent transport into the cell or accumulation of metabolites, such as glutamate. Secondly, mTORC1 regulates protein translation by controlling the phosphorylation of its downstream targets p70S6K and 4E-BP1 (Laplante and Sabatini, 2012). Protein supplementation induces the mTORC1 activation via two independent ways. Amino acids can be directly sensed by GTPases, amino acid transporters and receptors, which transmit the signal to mTORC1 by different signaling pathways (Zheng et al., 2016). This cascade occurs without modulation of PI3K and its downstream effector Akt. Additionally, branched chain amino acids induce the release of insulin, which act upon the PI3K-Akt-mTORC1 axis. Due to its vasodilatory effect, insulin also facilitates the uptake of amino acids from the blood circulation by the skeletal muscle cells. It is accepted that the availability of insulin plays a rather permissive effect, as it was shown that only ~10% of the post-prandial protein anabolism was dependent on insulin signaling, while ~90% was related to the increased amino acid levels (Volpi et al., 1996). Furthermore, Abdulla et al. (2016) reported that, in healthy adults, the positive effects of insulin *per se* on MPS became significant, only when the amino acid delivery to the skeletal muscle increased. At fixed amino acids concentrations, supraphysiological levels of insulin did not induce further increments in MPS (Greenhaff et al., 2008). To summarize, the beneficial effect of physiological hyperinsulinemia *per se* on MPS can only be observed as long as it concomitantly increases amino acid delivery and availability to the muscle (Fujita et al., 2006).

Among the amino acids, the essential amino acids (EAAs) (e.g., leucine) are the most effective to stimulate MPS (Stipanuk, 2007). Along with its stimulatory effect, leucine in particular also decreases MPB (Buse and Reid, 1975). Another mechanism ascribed to leucine involves its capacity to reverse insulin insensitivity in elderly. The precise mechanism(s) of the insulin sensitizing effects of long-term leucine supplementation are not fully elucidated yet, but may act through leucine-induced decreased adiposity, hepatic glucose production, hepatic steatosis, and adipose tissue inflammation (Zhang et al., 2007; Macotela et al., 2011; Binder et al., 2013). Insulin resistance, which often occurs with aging, is an important contributor to the pathophysiology of age-related sarcopenia, as this negatively affects mTORC1 signaling (Cleasby et al., 2016). Therefore, interventions that might positively affect the insulin sensitivity, are very likely to increase MPS (Børsheim et al., 2006; Solerte et al., 2008). Recently, a downstream metabolite of leucine, β-hydroxy β-methylbutyrate (HMB), gained much interest due to its broad ergogenic effects on muscle protein metabolism. HMB was shown to stimulate mTORC1 and the proliferation and differentiation of satellite cells (Cruz-Jentoft, 2017). Besides its anabolic actions, HMB was shown to exert anti-catabolic actions through the attenuation of proteasomal-mediated proteolysis and mitochondrial-mediated myonuclear apoptosis (Smith et al., 2005; Kovarik et al., 2010; Hao et al., 2011; Wilkinson et al., 2013).

Similar to resistance exercise, many elderly suffer from a blunted anabolic response to EAA intake. Therefore, it is generally suggested that larger doses may, at least partially, compensate for the blunted anabolic sensitivity (Paddon-Jones et al., 2004; Cuthbertson et al., 2005; Katsanos et al., 2006; Moore et al., 2015). Recent evidence-based recommendations state that 1.2–1.5 g.kg^−1^ BW should be ingested on a daily base by elderly, while the RDA for young adults is 0.8 g.kg^−1^ BW (Deer and Volpi, 2015; Devries and Phillips, 2015; Nowson and O'Connell, 2015; Moore et al., 2017). Furthermore, an intake of 25–30 g high quality proteins (~10 g EAAs) each meal has been proven to maximally stimulate MPS in elderly for 24 h (Deer and Volpi, 2015; Devries and Phillips, 2015; Nowson and O'Connell, 2015).

Despite the well-established positive findings of acute protein/leucine supplementation on the stimulation of MPS, the effectiveness of long-term protein supplementation as a treatment for age-related sarcopenia is more equivocal. On the one hand, a recent meta-analysis demonstrated the beneficial effects of leucine supplementation on MPS in elderly (be it without changes in lean body mass or lean leg mass) (Xu et al., 2015), while other studies failed to show a consistent effect of protein/leucine supplementation on muscle mass, strength and/or function (Bonnefoy et al., 2003; Verhoeven et al., 2009; Tieland et al., 2012b). The review of Hickson (2015) concluded that both whole-protein and EAA supplementation failed to show consistent effects on muscle mass, strength or function. Accordingly, it was suggested that, despite its acute anabolic effects, leucine supplementation has no beneficial effects on skeletal muscle mass or function on the long-term in muscle wasting conditions (Ham et al., 2014). In contrast to leucine, studies that focused on HMB yielded more consistent results (Hickson, 2015; Wu et al., 2015). Consequently, HMB might be more effective for the preservation of muscle mass, strength and functionality in elderly. However, more research is required to further elucidate the efficacy of the long-term intake of amino acids and their metabolites to combat age-related sarcopenia. In addition, it might be of great interest to develop alternative treatments that sensitize the skeletal muscle to leucine and anabolic stimuli in general, such as anti-inflammatory strategies.

### Combined protein supplementation and resistance exercise

Since both protein supplementation and resistance exercise are suggested to induce muscle hypertrophy by stimulating the mTORC1 signaling pathway, it is not surprising that their combined effect was broadly researched. In healthy adults, evidence indicates that the combined intervention induces additional gains in muscle mass and muscle strength, when compared to resistance training as such (Morton et al., 2017). In elderly, however, the findings are less conclusive, as many studies reported no superiority of resistance exercise with protein supplementation compared to resistance exercise as such (Fiatarone et al., 1994; Godard et al., 2002; Candow et al., 2006; Kukuljan et al., 2009; Verdijk et al., 2009; Denison et al., 2015; Thomas et al., 2016). Denison et al. (2015) suggested that an additional effect due to protein supplementation was mainly to be expected in subjects with low basal protein intakes, whereas those with adequate basal intakes would benefit less from additional protein supplementation. Since adequate protein intake is often problematic in frail and institutionalized elderly, it is of importance to not only emphasize on protein supplementation during resistance training, but also to ensure a sufficient basal protein intake in these subpopulations (Tieland et al., 2012a; Thomas et al., 2016).

## Inflammation-reducing approaches targeting age-related sarcopenia

**Figure 1:**
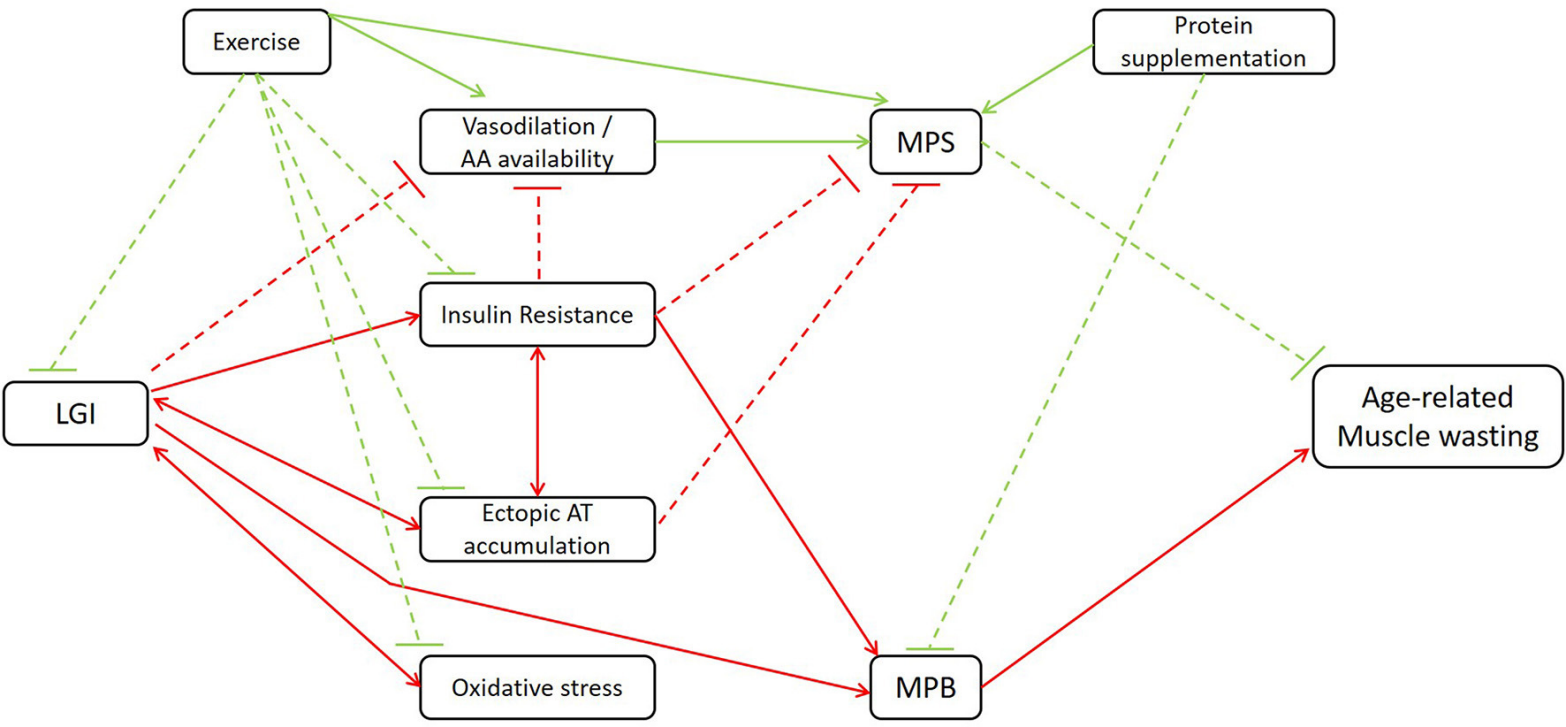
Schematic illustration of the mechanisms through which LGI may indirectly affect age-related muscle wasting. LGI, low-grade inflammation; AA, amino acid; AT, adipose tissue; MPS, muscle protein synthesis, MPB, muscle protein breakdown. Red lines contribute to the induction of muscle wasting; green lines to the attenuation of muscle wasting. Dashed lines: inhibitory signaling; full lines: stimulatory signaling. Additional to the association between LGI and age-related muscle wasting, the beneficial effects of classic strategies such as exercise and protein supplementation are illustrated.

As stated earlier, inflammation is closely involved in both the blunted anabolic response and increased catabolic processes in elderly. In the following section, nutritional strategies that attenuate muscle wasting in elderly, partially regulated through anti-inflammatory mechanisms, will be discussed. Figure 1 gives an overview of the mechanisms through which LGI may indirectly affect age-related muscle wasting.

### Vitamin D supplementation

Historically, vit D supplementation has been broadly applied in elderly, due to its well established effects on calcium (Ca^2+^) and bone homeostasis. It was shown that supplementation of vit D, preferably together with Ca^2+^, increases bone mineral density and decreases the risk of osteoporotic fractures in elderly. More recently, vit D has also been shown to play a regulatory role in metabolic pathways implicated in muscle wasting, and in the regulation of the immune system. It seems that vit D has beneficial effects on muscle strength (Muir and Montero-odasso, 2011; Beaudart et al., 2014), however, this only seems to apply to vit D-deficient elderly (Janssen et al., 2002; Stockton et al., 2011). In contrast, muscle mass seems not to be increased by vit D supplementation in elderly (Beaudart et al., 2014). Since the prevalence of vit D deficiency is very high among elderly (up to 42% in the U.S.), its supplementation should be recognized as an important intervention in elderly (Forrest and Stuhldreher, 2011).

The mechanisms through which vit D affects muscle cell functioning can be broadly divided into two categories, the genomic and non-genomic effects. For the genomic effects, binding of vit D to its nuclear receptor, a transcription factor, is required. This will result in changes in gene transcription, e.g., stimulation of cell proliferation and terminal differentiation, among others, both important in muscle development and growth (Boland, 1986; Ceglia and Harris, 2013). Indeed, decreases in muscle functioning related to vit D signaling with aging, can be mainly ascribed to the decreased vit D receptor expression in old skeletal muscle cells (Bischoff-Ferrari et al., 2004). Salles et al. (2013) also showed that vit D positively affected the expression of proteins involved in the insulin-Akt-mTORC1 pathway, along with an increased MPS in C_2_C_12_ muscle cells. These findings were recently confirmed *in vivo*, as vit D supplementation restored the blunted anabolic response in vit D-deficient old rats (Chanet et al., 2017). To our knowledge, these mechanisms were not yet studied in humans. The non-genomic “rapid” effects are mediated through the membrane-bound vit D receptor. Firstly, this regulates the influx of Ca^2+^ and inorganic phosphate in skeletal muscle cells, important for the regulation of muscle contractions and for the production of energy-rich phosphate groups, respectively. A second non-genomic effect involves the activation of protein kinase C. This kinase plays a crucial role in the regulation of MPS by mediating the anabolic signaling of both insulin (in an IRS1- and PI3K-dependent way) and leucine on protein synthesis (Selless and Boland, 1991; Mendez et al., 1997; Yagasaki et al., 2003).

As mentioned earlier, vit D also modulates the immune system and might therefore be an interesting target to combat LGI. Apart from muscle cells, the vit D receptor is expressed by cells which play a key role in immunity, such as macrophages and lymphocytes. Binding of vit D to its receptor in macrophages inhibits NF-κB activity (a transcriptional key regulator in the upregulation of pro-inflammatory mediators), and thereby attenuates TNFα production (Cohen-lahav et al., 2006). The same NF-κB-dependent inhibition of cytokine secretion through vit D signaling is present in lymphocytes (Calton et al., 2015). In murine models, vit D signaling also stimulates the production of lymphoid cell lineages with anti-inflammatory properties, such as Treg cells (Tian et al., 2015). Another mechanism involves a direct induction of relevant genes by vit D in specific liver cells, resulting in an elevated production of insulin-like growth factor-1 (IGF-1), which has previously been shown to engage in anti-inflammatory actions (Bellini et al., 2011; Yu et al., 2012; Wang et al., 2017). Furthermore, IGF-1 is also engaged as a stimulator of cell growth and proliferation through activation of the Akt signaling pathway.

Compared to rodent studies, findings from human interventions with vit D supplementation are very hard to interpret. Most human studies, which link vit D signaling to anti-inflammatory actions, focused on several diseases, each with their specific inflammatory context, which results in ambiguous evidence. Consequently, there is a need for human trials, which study the effects of vit D supplementation in elderly with limited comorbidities. In general, it can be concluded that vit D supplementation might be useful in elderly, as they belong to a population that is prone to vit D deficiency due to reduced exposure to sunlight, low oral intake of vit D, intestinal malabsorption, and decreased vit D hydroxylase activity in the kidneys.

### N-3 polyunsaturated fatty acids

N-3 poly unsaturated fatty acids (PUFAs) are characterized by a double bond at the third carbon from the methyl end of the carbon chain. Besides their role as a structural component, i.e., in membranes, PUFAs function in several cellular processes as regulatory or signaling molecules. In elderly, long-term n-3 PUFA supplementation increases muscle volume and muscle strength (Smith et al., 2015). Furthermore, n-3 PUFA/fish oil supplementation has additional beneficial effects on resistance training-induced muscle functionality in elderly women (Rodacki et al., 2012; Da Boit et al., 2017), though without changes in muscle mass (Da Boit et al., 2017). The anabolic effects of n-3 PUFAs can be ascribed to an increased MPS, mainly regulated through activation of the mTORC1 signaling pathway, as observed in both animal (Gingras et al., 2007; Wei et al., 2013) and human (Smith et al., 2011; Yoshino et al., 2016) studies. A possible link can be found with an increased insulin sensitivity, as it was shown that PUFA supplementation leads to increased phosphorylation of the insulin receptor and its downstream signaling (Kamolrat and Gray, 2013; Wei et al., 2013). Furthermore, enhanced insulin signaling induces vasodilation, which increases amino acid availability (Smith, 2016).

Besides their anabolic capacities, n-3 PUFAs possess well-established anti-inflammatory properties. Once transported into the cell, PUFAs are either stored, oxidized or incorporated into cellular membranes. PUFAs, released from the cell membranes, may form prostanoids, which are involved in direct inflammatory actions or regulate the production of other mediators such as inflammatory cytokines (Williams et al., 2000). When consuming a typical western diet, rather high rates of the n-6 PUFA arachidonic acid (ARA) are integrated in the membrane phospholipids of inflammatory cells, while proportions of the n-3 PUFA eicosapentaenoic acid (EPA) or docosahexaenoic acid (DHA) are low. Therefore, ARA is often the main substrate for the formation of prostanoid synthesis (Bagga et al., 2003). After mobilization from the membrane phospholipids by the enzyme phospholipase A_2_, ARA binds the cyclooxygenase enzymes (COX-1/2) from which downstream intermediates are further metabolized to finally synthesize pro-inflammatory prostanoids. When more n-3 PUFAs are integrated in the diet, less n-6 PUFA-derived pro-inflammatory prostanoids will be formed.

Early findings indicate the association between muscle protein metabolism and a certain subclass of the prostanoids, i.e., prostaglandins (Rodemann and Goldberg, 1982; Rodemann et al., 1982). The research group of Goldberg observed that PGE_2_, produced by muscle upon ARA incubation, consistently stimulated protein degradation (+22%), while no significant changes in MPS were observed. PGF_2α_, derived from ARA, contrarily, had no effect on MPB but increased MPS. These findings are of great interest from an aging perspective, as an increased LGI was related to an overexpression of COX-2, among others, eventually evoking a pro-inflammatory status (e.g., elevated levels of PGE_2_) (Chung et al., 2006). More recently, Standley et al. (2013) suggested that COX-induced expression of PGE_2_ increased the expression of IL-6 and MuRF-1, which are both related to MPB and might therefore explain the mechanism through which PGE_2_ is linked to muscle proteolysis.

Despite promising associations between n-3 PUFAs and muscle metabolism, this area of research needs to be further explored. Animal studies revealed that the EPA was effective in attenuating the ubiquitin-proteasome pathway in several muscle wasting conditions, such as fasting, cachexia and arthritis (Whitehouse and Tisdale, 2001; Whitehouse et al., 2001; Castillero et al., 2009). Furthermore, in obese adults, with elevated inflammatory mediators (e.g., IL-6), n-3 PUFAs were effective in alleviating systemic inflammation (Itariu et al., 2012; Allaire et al., 2016). It would be of interest to expand this knowledge to elderly suffering from LGI in order to develop strategies that support/optimize the current interventions. Recently, a paper confirmed that n-3 PUFA supplementation in elderly downregulated pathways related to calpain- and ubiquitin-mediated proteolysis in skeletal muscle (Yoshino et al., 2016). This shows the anti-proteolytic potential of n-3 PUFAs in age-related muscle wasting.

### The COX-pathway and muscle metabolism

There seems to be an interesting link between inflammation and muscle protein metabolism. A lower MPS was reported in old rats suffering from LGI (Balage et al., 2010) and their MPS was increased when the LGI was blunted by NSAID administration (Marzani et al., 2008; Rieu et al., 2009). Interestingly, LGI also impaired muscle protein anabolism in response to resistance training in elderly (Trappe et al., 2002). As mentioned before, these effects might be related to certain intermediates of the COX pathway, i.e., prostaglandins. COX-inhibiting interventions through NSAID administration might therefore be effective in improving muscle protein metabolism and thus for the treatment of muscle wasting (Greig et al., 2009). This idea was further strengthened by the cross-sectional study of Landi et al. (2013), which investigated the relationship between NSAID use and sarcopenia in community-dwelling elderly (>80 years). In this study only 9% of the subjects that chronically used NSAIDs were affected by sarcopenia, compared to 32% in the non-user group (Landi et al., 2013). Although it tantalizing to promote daily consumption of NSAIDs to overcome the age-related muscle anabolic resistance, the associated risks, such as gastric, cardiovascular and hepato-renal adverse events, cannot be denied (Greig et al., 2009). In this perspective, other anti-inflammatory compounds might be of great interest in the search for a physiological-holistic approach to combat age-related sarcopenia. It should be noted though that anti-inflammatory compounds, inhibiting COX-2 and concomitant PG synthesis, might negatively affect skeletal muscle regeneration and hypertrophy. Some studies reported that COX-2 inhibition resulted in a decreased size of regenerating muscle fibers following injury and negatively affected *ex vivo* satellite cell proliferation, differentiation and fusion (Bondesen et al., 2004; Mendias et al., 2004). Furthermore, COX-2 inhibition decreased intramuscular macrophage accumulation and cell proliferation, and the consequent hypertrophic response in a synergist ablation model (Novak et al., 2009). Future studies should clarify whether these inhibitory effects on muscle regeneration also apply to sarcopenic elderly, and/or whether nutritional anti-inflammatory strategies can enforce the positive effects of classic strategies.

## Conclusion

Plural mechanisms were shown to contribute to the etiology and/or progression of muscle wasting with advancing age. Somehow, many of these mechanisms interfere with inflammatory mediators. However, further research is required to determine through which mechanisms inflammation directly or indirectly affects MPB and MPS with aging. Classic interventions such as protein supplementation and resistance exercise are generally accepted to be the most appropriate to positively affect muscle protein metabolism in elderly. However, not all studies univocally support the effectiveness of these strategies for long-term treatment of age-related muscle wasting. Elderly, and very old/frail seniors in particular, might benefit from a strategy which primarily focusses on alleviating their muscle insensitivity to anabolic stimuli. In this regard, the treatment of LGI in these elderly might play an important role. Given the limited applicability of NSAIDs, other (non-pharmaceutical) approaches to attenuate LGI should gain more attention. Additionally, future research should also focus on possible interactions with the “classical” anabolic treatments, in order to develop a holistic approach, that takes into account the personal capabilities and experiences of the old individual.

## Author contributions

Substantial contributions to the conception or design of the work: SD, LR, KK. Drafting the work or revising it critically for important intellectual content: SD, LR, KK. Final approval of the version to be submitted: SD, LR, KK. All authors agree to be accountable for all aspects of the work.

### Conflict of interest statement

The authors declare that the research was conducted in the absence of any commercial or financial relationships that could be construed as a potential conflict of interest.
